# The Comorbid Relationship Between Migraine and Asthma: A Systematic Review and Meta-Analysis of Population-Based Studies

**DOI:** 10.3389/fmed.2020.609528

**Published:** 2021-01-13

**Authors:** Long Wang, Zi-Ru Deng, Mei-Dan Zu, Juan Zhang, Yu Wang

**Affiliations:** ^1^Department of Neurology, The First Affiliated Hospital of Anhui Medical University, Hefei, China; ^2^Department of Neurology, The Second People Hospital of Hefei, Hefei, China

**Keywords:** comorbid, migraine, asthma, risk, meta-analysis

## Abstract

**Objective:** Recent studies have indicated a pathophysiologic link between migraine and asthma. This meta-analysis aimed to comprehensively estimate the risk ratio for migraine in asthma as well as that of asthma in migraine based on available evidence.

**Method:** We systematically searched the electronic databases including PubMed, Web of Science, and SCOPUS for population-based studies that measured either the odds or the risk of asthma in subjects with migraine as well as that of migraine in subjects with asthma. The titles and abstracts were screened by two independent reviewers to identify eligible studies, and this was followed by full-text review of the included studies. Newcastle–Ottawa Scale (NOS) was used to assess the risk of bias of included literature. A meta-analysis was conducted with Review Manager 5.3 Software to calculate the odds ratio (OR) for case-control and cross-sectional studies and either relative ratio (RR) or hazard ratio (HR) for cohort studies, and the source of heterogeneity was assessed. Subgroup and sensitivity analyses were conducted, and the I^2^ test were used to assess the source of heterogeneity. The funnel plot, Galbraith plot, and Egger's test were used to evaluate publication bias.

**Results:** Fifteen published studies covering a total of 1,188,780 individuals were identified. Pooled analysis indicated that migraine was associated with increased odds (OR = 1.54; 95% CI: 1.34~1.77) and risk for asthma (HR = 1.42; 95% CI: 1.26~1.60), and asthma associated with increased odds (OR = 1.45; 95% CI: 1.22~1.72) and risk for migraine (HR = 1.47; 95% CI: 1.41~1.52).

**Conclusion:** Migraine is a potential risk indicator for asthma, and *vice versa*, asthma is a potential risk indicator for migraine. However, future prospective cohort studies are warranted to provide more evidence concerning the detailed association between migraine and asthma.

## Key Messages

This is the first meta-analysis of population-based studies that assessed the comorbid relationship between migraine and asthma. Migraine is a potential risk indicator for asthma, and *vice versa*, asthma is a potential risk indicator for migraine.

## Introduction

Asthma is a common airway disorder with clusters of respiratory symptoms including recurrent episodes of coughing, shortness of breath, and dyspnea (1). Epidemiological investigation revealed that the incidence of asthma varies widely among different countries ranging from ~7 to 18% (2). Although its condition can be alleviated by inhaled corticosteroid (ICS) or by combined treatment with ICS and long-acting β-agonists (LABA) (3), there is no specific drug that can permanently prevent the recurrence of an asthma attack. Thus, the current goal of asthma management is to focus on managing rather than curing the disease (4). In recent years, increasing attention has been paid to the comorbid conditions of asthma (5–8), which cause a significant burden on families and health care systems (9).

Migraine is a common chronic neurological disorder characterized by multiple symptoms of headache, nausea, vomiting, and/or aura. Research data from Global Burden of Disease (GBD) have shown that as many as 1.04 billion people suffered from migraine globally (10), and its comorbidities contribute to the overall burden of migraine and further lower quality of life (11).

Migraine is frequently comorbid with asthma, and this condition has been recognized as “acephalic migraine” and “pulmonary migraine” (12–14). The common pathophysiologic mechanisms of inflammation and immune dysfunction between the two diseases could explain the bidirectional association. For example, atopy, various inflammatory mediators, or elevated neuropeptide mediators contribute to both migraine and asthma (15). In addition, the shared triggering and environmental factors such as air pollutants are related to the occurrence or aggravation of asthma and the rate of emergency department visits for migraine (16, 17). Moreover, emotional stress or psychological distress, such as anxiety or depression, due to asthma could increase an organism's susceptibility to migraine and *vice versa* (18).

Whereas, the exact bidirectional association between the two diseases was still unclear through genetic interaction, shared environmental triggering factors have been reported among migraine and asthma. A fundamental step in exploring the association between asthma and migraine is to understand the strength of the comorbid relationship between the two diseases. Therefore, we conducted a systematic review and meta-analysis of population-based evidence to determine the risk of asthma associated with migraine and *vice versa*.

## Methods

Our meta-analysis was conducted on the basis of the Preferred Reporting Items for Systematic reviews and Meta-Analyses (PRISMA) guidelines (19).

### Search Strategy

Two investigators (Long Wang and Zi-Ru Deng) independently searched published articles indexed in PubMed, Web of Science, and SCOPUS database until March 2020. Text words and Medical Subject Headings (MESH) terms used were (headache OR migraine) AND (asthma) NOT (“case report” OR review OR “meta-analysis”).

### Study Selection

We included population-based studies that met the following inclusion criteria: (1) studies with design being population-based cohort, case-control, cross-sectional literatures; (2) the exposed group consisted of individuals with migraine or asthma, and the control group consisted of people without migraine or asthma; (3) the outcomes used were odds ratio (OR), relative risk (RR), or hazard ratio (HR) to assess the comorbid relationship between migraine and asthma. Studies were not limited by language of publication. If more than one article reported data derived from one cohort or one health database, only the longest follow-up and more comprehensive study was included. We excluded cases series, letters without original data, duplicate studies, and reviews as they did not satisfy the eligibility criteria.

### Data Extraction and Quality Assessment

One reviewer extracted the data using a standardized data collection form, and the findings were checked by another reviewer. The following pieces of information were extracted from the included studies: surname of the first author, year of publication, country of study, study design, study subjects, age of patients with migraine or asthma, diagnostic criteria, years of follow-up, outcome, and adjustment. The Newcastle–Ottawa Scale (NOS) was used to assess the quality of the studies (20). For case-control and cross-sectional studies, we assessed the following three domains: (1) selection of the studies: adequacy of the case definition, representativeness of the cases, definition of the controls, and selection of the controls; (2) comparability: comparability of cases and controls based on the design or analysis; (3) exposure: ascertainment of exposure, same method of ascertainment of cases and controls, and non-response rate. The domains evaluated for cohort studies included: (1) selection of the studies: representativeness of the exposed cohort, selection of the control cohort, ascertainment of exposure, and demonstration that outcome was not present at the beginning of the study; (2) comparability: comparability of exposure and non-exposure cohorts; (3) outcome: evaluation of outcome, adequate length of follow-up, and adequacy of follow-up cohorts. A study would be rated as uncertain, low, or high risk based on its effects on validity for each domain.

### Data Synthesis and Analysis

Data analysis was performed using Review Manager 5.3 Software. The random-effects model for meta-analysis was conducted to pool the OR of migraine in asthma as well as OR of asthma in migraine. We extracted the adjusted HR and 95% CI from cohort studies to assess the comorbid relationship between migraine and asthma. I^2^ was calculated to quantify the heterogeneity among the included studies. A value of I^2^ of 0–25% represents absence of heterogeneity; 25% ≤ I^2^ < 50%, low heterogeneity; 50% ≤ I^2^ < 75%, moderate heterogeneity; I^2^ ≥ 75%, substantial heterogeneity (21). Subgroup and sensitivity analyses were conducted to check potential sources of bias among studies. The funnel plot, Galbraith plot, and Egger's test were used to evaluate publication bias.

## Results

### Characteristics of Included Studies

**Figure 1:**
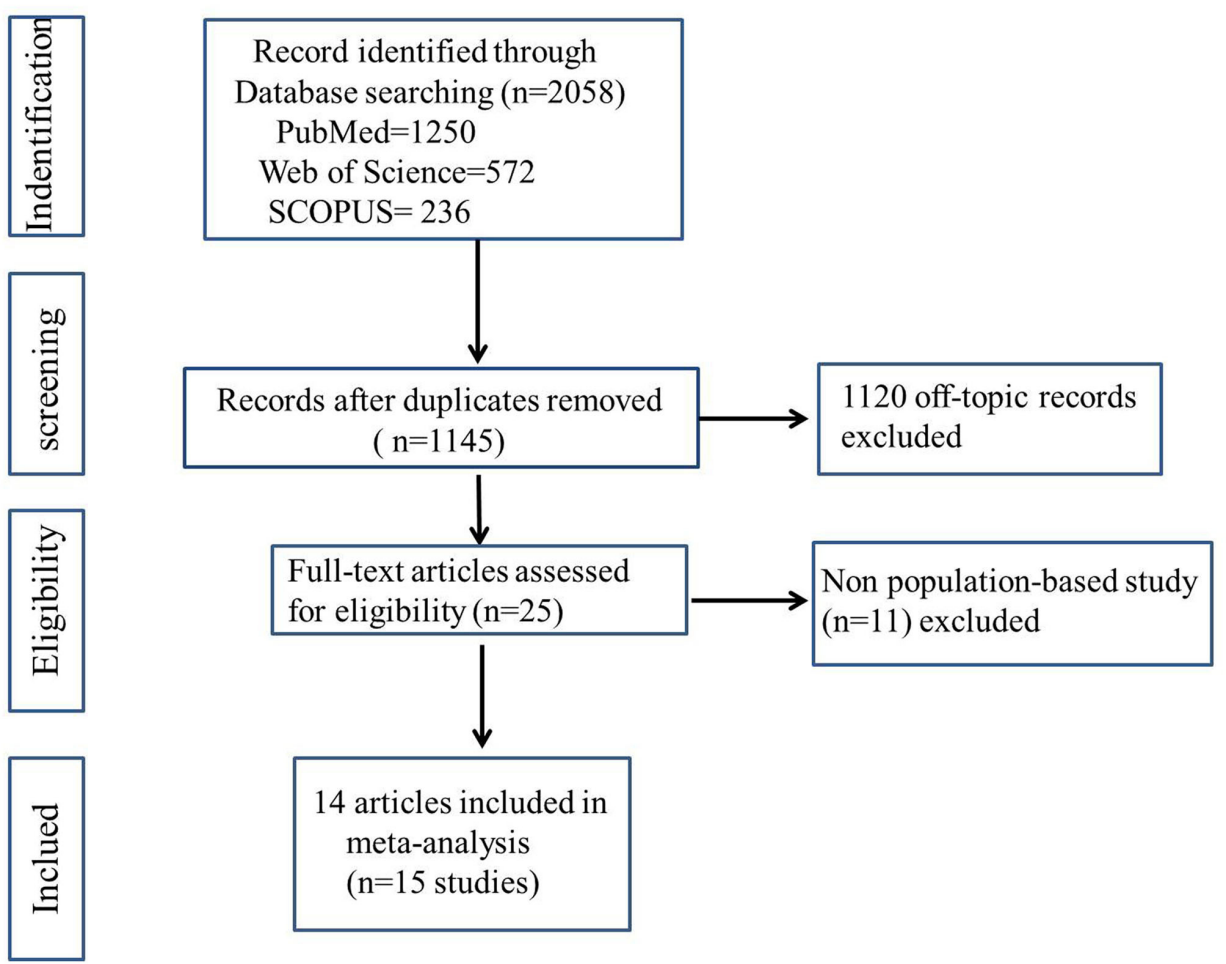
Flowchart of study identification for meta-analysis.

As illustrated in Figure 1, we retrieve a total of 15 studies from the database search after removing duplicates. Eventually, 14 articles were included in our meta-analysis, one of which described two separate studies, resulting in a total of 15 studies, which comprised a total of 1,188,780 subjects. The 2019 article by Kim et al. (22) reported two cohort studies (both migraine and asthma exposure).

The characteristics of the included studies are shown in Table 1. Of 15 studies, there were a total of three case-control, six cross-sectional, and six cohort studies. Of these articles included in the final meta-analysis, one is from the United Kingdom (23), three from the USA (11, 24, 25), two from Korea (18), four from China (9, 26–28), one from Denmark (29), one from Norway (30), one from Israel (31), and two from Sweden (32, 33). Majority of studies examined adults, while three examined primarily children or adolescents. Five studies relied upon questionnaire or medical records to identify cases of migraine and/or asthma, while nine relied upon validated International Classification of Headache Disorders (ICHD) or International Classification of Diseases (ICD) codes (34, 35).

**Table 1:** The characteristics of the included population-based studies in this meta-analysis.

**First author, year**	**Country**	**Study design**	**Sample size**	**Target group**	**Age (years)**	**Diagnosis of migraine/asthma**	**Follow-up years**	**Outcome**	**Confounders adjusted**
**Asthma in patients with migraines or non-migraines**									
Buse DC, 2020	US	Prospective, longitudinal cross-sectional	Total: 92,586 Migraine: 15,133 No migraine: 77,453	Adult	Migraine: 43 ± 13.6 No migraine: 52 ± 15.7	ICHD-2	NA	OR: 2.03 (1.93, 2.14)	Age, gender, Hispanic origin, race, marital status, employment, household income
Kim SY, 2019	Korea	Observational cohort study	Total: 180,220 Migraine: 36,044 No migraine: 144,176	Adult	≥20	ICD-10	3.6	HR: 1.37 (1.33, 1.41)	Age, sex, income, and region of residence
Wei CC, 2018	Taiwan	National case-control	Total: 80,650 Migraine: 16,130 No migraine: 64,520	Adolescents 7–18	Migraine: 13.6 ± 3.15 No migraine: 13.6 ± 3.16	ICD-9	NA	OR: 1.76 (1.66, 1.87)	NA
Peng YH, 2018	Taiwan	Retrospective cohort study	Total: 33,235 Migraine: 6,647 No migraine: 26,588	Adult	Migraine: 40.1 ± 10.2 No migraine: 39.8 ± 10.4	ICD-9	12	HR: 1.37 (1.16, 1.60)	Sex, age, occupational status, insurance premium, urbanization, comorbidities, and annual number of OPD visits
Lateef TM, 2012	US	Cross-sectional	Total: 6,207 Migraine: 545 No migraine: 5,662	Adolescents	13–18	ICHD-II	NA	OR: 1.25 (1.0, 1.56)	Age, sex, and ethnicity
Czerwinski S, 2012	USA	Cross-sectional	Total: 3,731 Migraine: 670 No migraine: 3,061	Adult	Migraine: 32.5 ± 4.6 No migraine: 32.7 ± 4.5	Questionnaire and medical records	NA	OR: 1.38 (1.09, 1.74)	Maternal age, parity, marital status, history of chronic hypertension, and pre-pregnancy body mass index
Chen CY, 2012	Taiwan	Retrospective cohort study	Total: 4,738 Migraine: 948 No migraine: 3,790	Adult	20~59	ICD-9	1	RR: 2.27 (1.59, 3.25)	Age, gender, urbanization level of the residence, income, and hospital setting
Le H, 2011	Denmark	Cross-sectional	Total: 31,671 Migraine: 7,991 No migraine: 23,680	Adult	44 average	Questionnaire	NA	OR: 1.36 (1.18, 1.56)	NA
Becker C, 2008	Sweden	Cohort study	Total: 103,376 Migraine: 51,688 No migraine: 51,688	Adult	30–79	Medical records	until asthma was diagnosed	RR: 1.3 (1.1, 1.4)	NA
Aamodt AH, 2007	Norway	Cross-sectional	Total: 51,383	Adult	≥20	IHS	NA	OR: 1.5 (1.3, 1.7)	NA
Davey G, 2002	UK	Case-control	Total: 129,356 Migraine: 64,678 No migraine: 64,678	Adult	30–50	OXMIS diagnostic codes	NA	OR: 1.59 (1.54, 1.65)	NA
**Migraine in patients with asthma or non-asthma 471,627**									
Kim SY, 2019	Korea	Cohort study	Total: 227,118 Asthma: 113,059 No asthma: 113,059	Adult	≥20	ICD-10	6.8	HR: 1.47 (1.41, 1.53)	Age, sex, income, and region of residence
Graif Y, 2018	Israel	Cross-sectional study	Total:113,671 Asthma: 4,581 No asthma: 109,090	Adolescents	17 average	Questionnaire and medical records	NA	OR: 1.42 (1.19, 1.68)	Sex, allergic rhinitis, gender, immigrant, obese, overweight, ≤3 siblings, urban residence
Tsiakiris G, 2017	Sweden	Cross-sectional study	Total: 3,040 Allergic asthma:164 No allergic asthma: 2,876	Adult	Asthma: 48.1 ± 17.8 No asthma: 51.7 ± 16.8	Questionnaire	NA	OR: 1.87 (1.01, 3.48)	Sex and education
Peng YH, 2016	Taiwan	Cohort study	Total: 127,798 Asthma:25,560 No asthma: 102,238	≥12	Asthma: 51.4 ± 19.6 No asthma: 51.1 ± 19.6	ICD-9	Average 8.4	HR: 1.45 (1.33, 1.59)	Sex, age, the Charlson Comorbidity Index, common medications prescribed for patients with asthma, and annual outpatient department visits

*NA, not available; OR, odds ratio; RR, relative ratio; HR, hazard ratio; ICHD, The International Classification of Headache Disorders; ICD, The International Classification of Diseases*.

### Quality of Included Studies

NOS was used to assess the quality of the included studies. Of 15 studies included in the meta-analysis, 10 studies were of high quality (NOS score >8); five studies were of moderate quality (NOS score between 6 and 7) (See Supplementary Material 1).

### Risk of Bias Assessment

**Figure 2:**
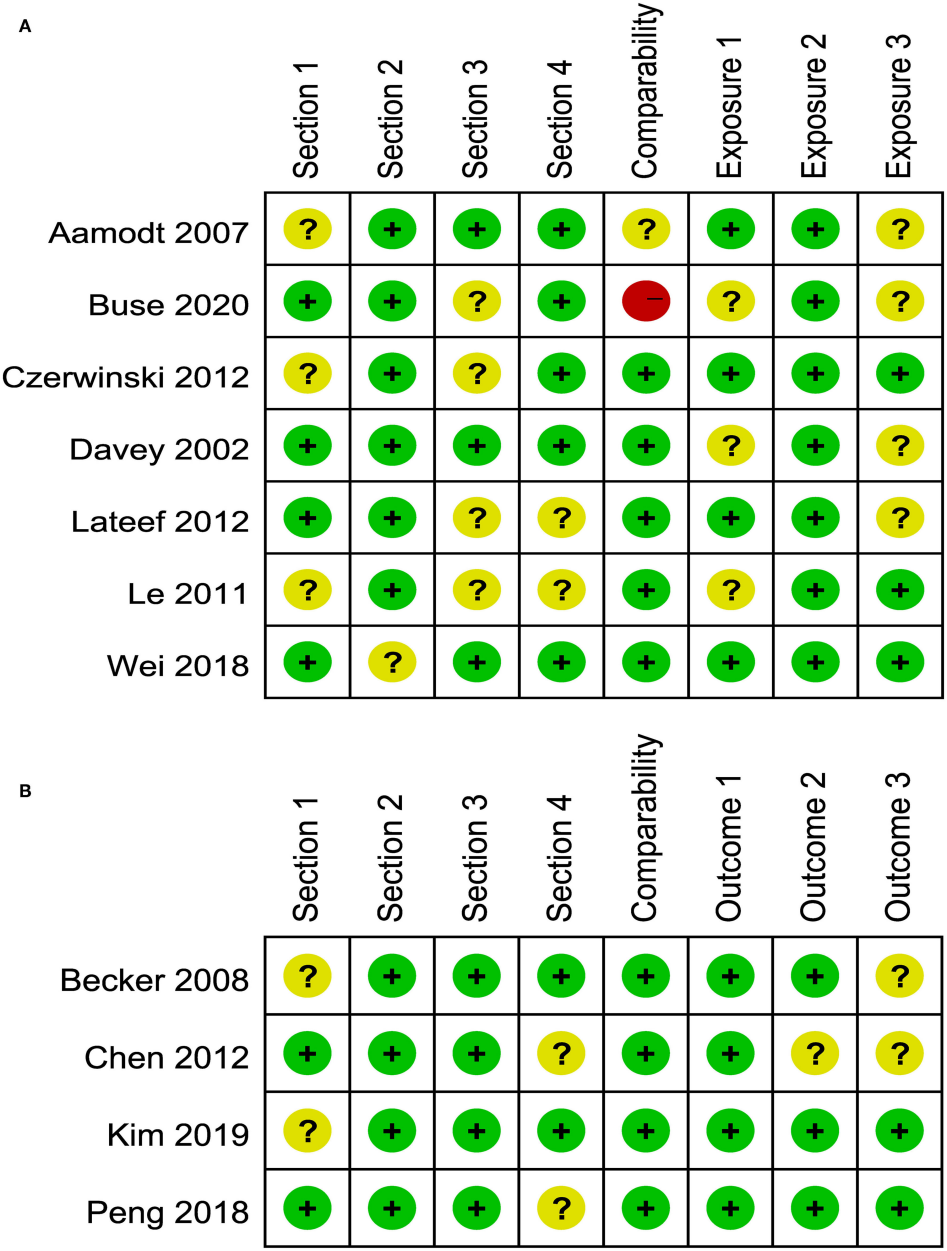
**(A)** Risk of bias of included case-control and cross-sectional studies. **(B)** Risk of bias of included cohort studies.

**Figure 3:**
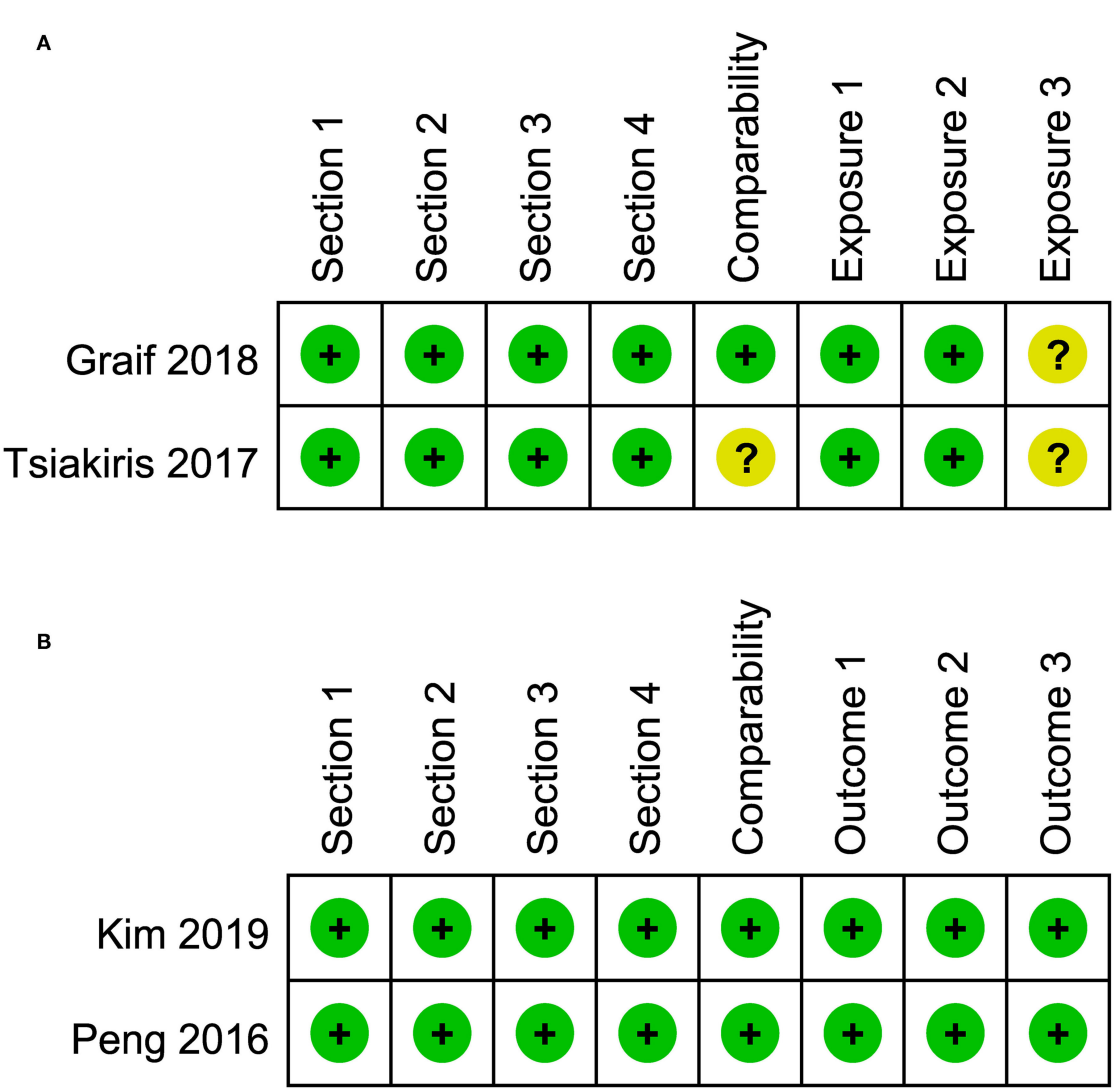
**(A)** Risk of bias of included cross-sectional studies. **(B)** Risk of bias of included cohort studies.

The risk of bias assessment for included studies is illustrated in Figures 2, 3. A number of the case-control or cross-sectional studies were rated as “uncertain risk of bias” for the different domain attributing to lack of description on confirmation of the corresponding content. Additionally, the 2020 study by Buse et al. (11) was rated as “high risk of bias” in comparability because age, sex, and ethnicity were unmatched in case group and control group.

### Odds of Asthma Among Migraine in Case-Control and Cross-Sectional Studies

**Figure 4:**
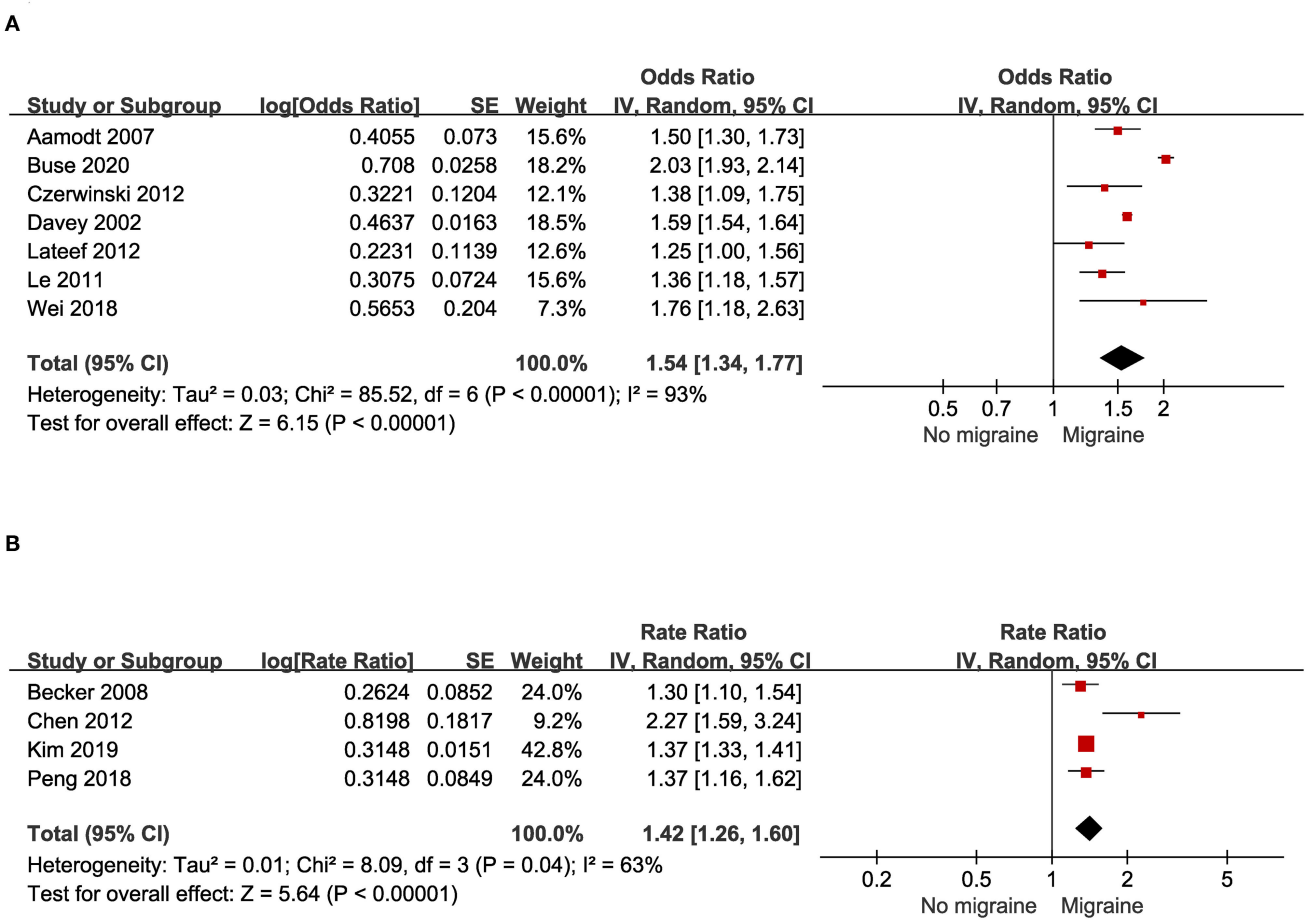
**(A)** Odds of asthma in patients with migraine in cross-sectional and case-control studies. **(B)** Risk of asthma in patients with migraine in cohort studies.

A total of seven studies, including three case-control studies and four cross-sectional studies with 495,229 migraine patients and control provided data for this outcome. As illustrated in Figure 4A, substantial heterogeneity was found (I^2^ = 93%). Migraine was associated with a significant increase of prevalent asthma (pooled OR: 1.54; 95% CI: 1.34~1.77).

### Migraine and Risk of Asthma in Cohort Studies

Four cohort studies with a total of 221,924 migraine patients and control provided data for this outcome. As illustrated in Figure 4B, substantial heterogeneity was found (I^2^ = 63%). People with asthma had a significantly increased risk for migraine (pooled RR: 1.42; 95% CI: 1.26~1.60).

### Odds of Migraine Among Patients With Asthma in Case-Control and Cross-Sectional Studies

**Figure 5:**
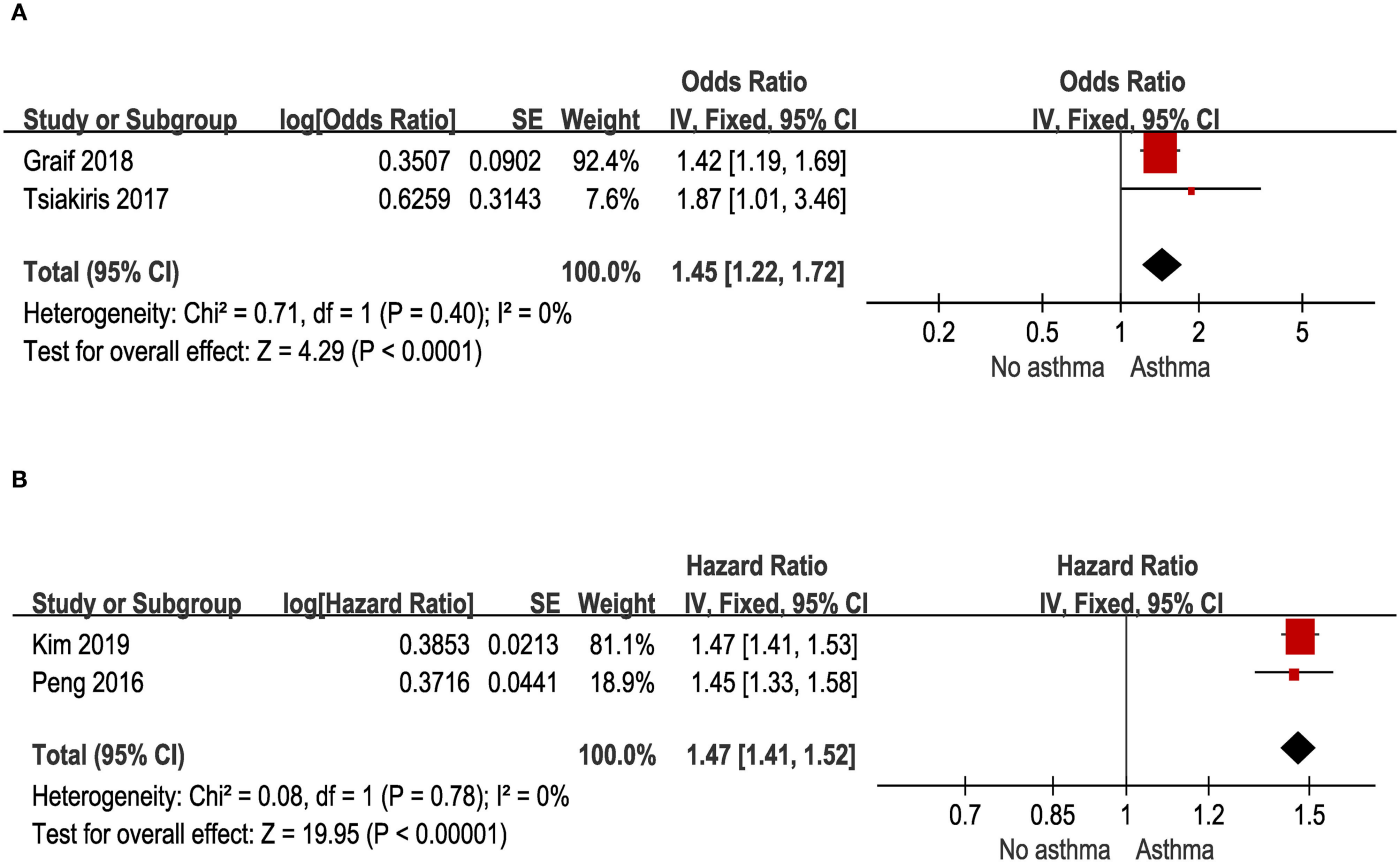
**(A)** Odds of migraine in patients with asthma in cross-sectional and case-control studies. **(B)** Risk of migraine in patients with asthma in cohort studies.

Two cross-sectional studies with a total of 116,711 asthma patients and control provided data for this outcome. As illustrated in Figure 5A, migraine was associated with a significant increase of prevalent asthma (pooled OR: 1.45; 95% CI: 1.22~1.72).

### Asthma and Risk of Migraine in Cohort Studies

Two cohort studies with a total of 354,916 asthma patients and control provided data for this outcome. As illustrated in Figure 5B, people with asthma had a significantly increased risk for migraine (pooled HR: 1.47; 95% CI: 1.41~1.52).

### Subgroup and Sensitivity Analyses

In the subgroup analysis for the OR of asthma in patients with migraine, studies were stratified by sample size, age, and location (Table 2). Heterogeneity was relatively lower both in the subgroups of sample size <50,000 (I^2^ = 42%) and adolescent (I^2^ = 53%). However, the heterogeneity was significant for both studies that assessed the OR in European (I^2^ = 98%) and Asian (I^2^ = 90%).

**Table 2:** Subgroup analysis for the odd ratio of asthma in patients with migraine.

**Subgroups** **studies**	**Included**	**OR (95% CI)**	**Heterogeneity (I^2^,** **Tau^2^, and *P*-value)**		
			**I^**2**^ (%)**	**Tau^**2**^**	***P*-value**
**Sample size**					
≥50,000	6	1.55 (1.34~1.79)	98.0	0.03	<0.001
<50,000	5	1.42 (1.24~1.62)	42.0	0.01	0.08
Age					
Adult	9	1.51 (1.34~1.70)	97.0	0.03	<0.001
Adolescent	2	1.42 (1.03~1.97)	53.0	0.03	0.03
**Regional distribution**					
Europe	5	1.44 (1.09~1.89)	98.0	0.09	0.009
Asia	6	1.52 (1.37~1.69)	90.0	0.01	<0.001

**Figure 6:**
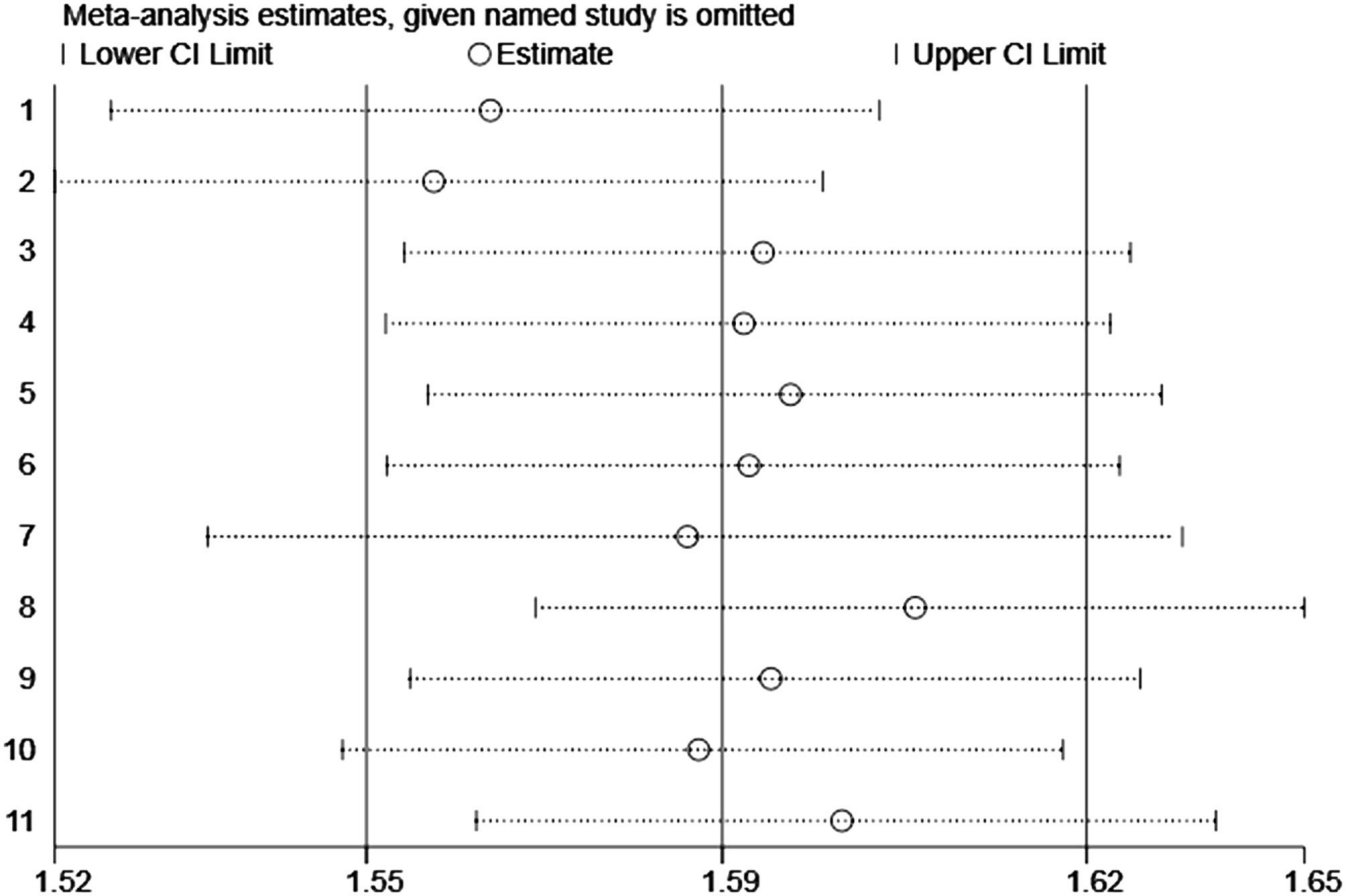
Plot of sensitivity analysis by excluding one study each time and the pooling estimate for the rest of the studies.

Moreover, we performed a leave-one-out sensitivity analysis to further explore the data heterogeneity; thus, none of the single studies significantly affected the pooled-effect size (Figure 6).

### Publication Bias

**Figure 7:**
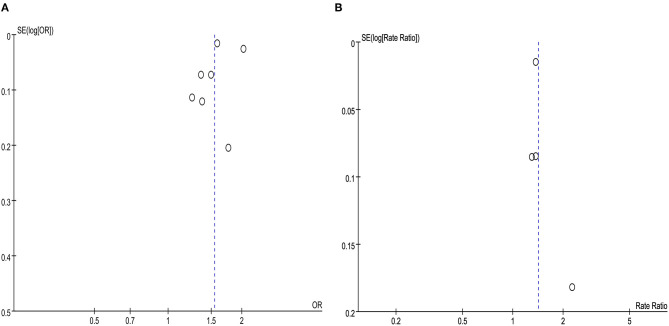
**(A)** Funnel plot for the association between migraine and risk of asthma in cross-sectional and case-control studies. **(B)** Funnel plot for the association between migraine and risk of asthma in cohort studies.

**Figure 8:**
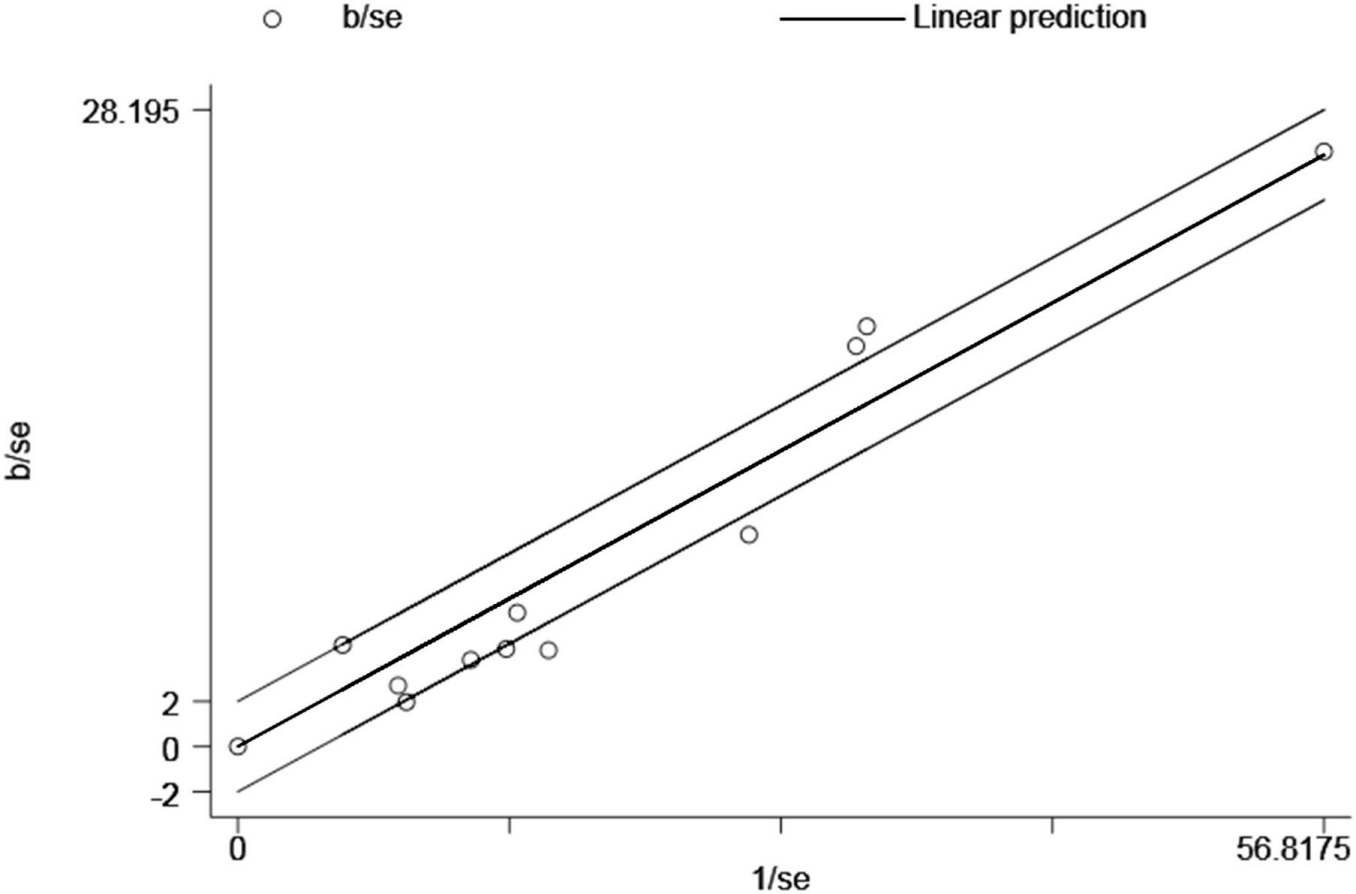
Galbraith plot for the association between migraine and risk of asthma.

For the overall meta-analysis of the relative risk of asthma among people with migraine, a visual inspection of the funnel plot (Figure 7), Galbraith plot (Figure 8), and Egger's tests also revealed no potential publication bias (Figure 7A, *P* = 0.476; Figure 7B, *P* = 0.531).

## Discussion

In this meta-analysis of population-based studies, we compiled current evidence on the association between migraine and asthma. We found that migraine was associated with 54% increased prevalence and 42% greater risk of asthma, and *vice versa*, asthma associated with 45% increased prevalence and 47% greater risk of migraine.

One previous review investigated the relationship between migraine and asthma with only two cohort studies and six case-control/cross-sectional studies, and respective risks of developing migraine or asthma were not analyzed based on the findings (36). By contrast, in the current analysis, we included and added more recent studies including three cohort studies, two case-control/cross-sectional studies, and two population-based studies that were left out, then separately analyzed data depending on the type of study design, thus providing robust evidence on the bidirectional association between migraine and asthma.

The exact pathway leading to comorbidity of migraine and asthma remains unclear. Previous studies have demonstrated an insight into the relationship between migraine and asthma from shared inflammatory, immune, and genetic factors (37–39). First, the bidirectional association between migraine and asthma is considered to be attributed to the common pathophysiological process of inflammation and immune dysfunction (39). Studies have shown that allergic responses triggered by allergens, such as pollen, specific grass, release various inflammatory mediators or elevated neuropeptide mediated by mast cells, leading to hypersensitivity of airway responses in patients with asthma (40). In migraine, mast cells in the dura matter activated by allergens may secrete pro-inflammatory and vasodilatory molecules, which are presumed to cause the activation of the trigeminal pain pathway underlying migraine pathogenesis (41, 42). In addition, parasympathetic hyperactivity is believed to be involved in the connection between asthma and migraine (43). An increased cholinergic tone has been suggested to trigger bronchospasm in asthma (44). Acetylcholine released by parasympathetic afferents can directly activate trigeminal pain pathway or provoke the degranulation of mast cell in meninges among migraine (45). Furthermore, transient receptor potential vanilloid subfamily member 1 (TRPV1) expressed in membranes of sensory afferent fibers has been shown to play a key role in the pathophysiology of both migraine and asthma (46, 47). TRPV1 expressed on the trigeminal nociceptors can be activated by various chemical substances, acid, and heat, which results in the release of various neuropeptides that cause neurogenic inflammation and exert a vasodilatory effect, both of which are crucial factors in the generation of migraine (47, 48). Similarly, TRPV1 expressed in airway C-fiber afferent neurons can be activated by endogenous activators or inhaled irritants, which has been observed in asthma patients (49, 50).

For the subgroup analysis, it is indicated that the source of heterogeneity may be due to the sample size and age. Therefore, more studies with large samples and different age groups are needed to clarify the bidirectional relationship between the two diseases.

To our knowledge, this is the first meta-analysis of population-based studies that assessed the comorbid relationship between migraine and asthma. Nevertheless, this meta-analysis has the following limitations. It is difficult to verify the causal relationship between migraine and asthma because there are only four and two cohort studies in the corresponding subgroup, respectively. In addition, as previously mentioned, though migraine and asthma may share a pathogenesis process, it is difficult to confirm whether there is a direct relationship between the two diseases. Moreover, only one case-control study was primarily of adolescents and five of six cohort studies were carried out in Asia, potentially limiting the generalizability of our findings to region and population. Even so, our findings are still of important implications. The bidirectional relationship we found might aid in preventing or identifying people with these two diseases. It is necessary to get further information about potential overlapping pathways in pathogenesis, which may facilitate the development of new treatment strategies for migraine and asthma.

## Conclusions

We found that migraine is a potential risk indicator for asthma, and *vice versa*, asthma is also a potential risk indicator for migraine. To further examine this finding and establish a more robust result, more large-scale prospective cohort studies are warranted to provide more evidence concerning the detailed association between migraine and asthma.

## Data Availability Statement

The original contributions generated for this study are included in the article/Supplementary Material, further inquiries can be directed to the corresponding author/s.

## Author Contributions

LW and Z-RD contributed to the data curation. LW contributed to the formal analysis, funding acquisition, and writing of the original draft. LW, JZ, and M-DZ contributed to the investigation, methodology, resources, and validation. Z-RD and YW contributed to the project administration. LW, Z-RD, and M-DZ acquired the software. LW and JZ contributed to the supervision and writing, review, and editing. All authors contributed to the article and approved the submitted version.

## Conflict of Interest

The authors declare that the research was conducted in the absence of any commercial or financial relationships that could be construed as a potential conflict of interest.

## References

[1] Mims JW. Asthma: definitions and pathophysiology. Int Forum Allergy Rhinol. (2015) 5(Suppl. 1):S2–6. 10.1002/alr.21609 26335832

[2] Akinbami LJ Rossen LM Fakhouri TH I Simon AE Kit BK. Contribution of weight status to asthma prevalence racial disparities, 2–19 year olds, 1988–2014. Ann Epidemiol. (2017) 27:472–8. 10.1016/j.annepidem.2017.07.004 28778655 PMC6413506

[3] O'Byrne PM Pedersen S Lamm CJ Tan WC Busse WW Severe exacerbations and decline in lung function in asthma. Am J Respir Crit Care Med. (2010) 182:983–4. 10.1164/ajrccm.182.7.983a 18990678

[4] Peng YH Chen KF Kao CH Chen HJ Hsia TC Chen CH Liao WC. Risk of migraine in patients with asthma: a nationwide cohort study. Med. (Baltimore). (2016) 95:e2911. 10.1097/MD.0000000000002911 26945388 PMC4782872

[5] Boulet LP Boulay MÈ. Asthma-related comorbidities. Expert Rev Respir Med. (2011) 5:377–93. 10.1586/ers.11.34 21702660

[6] Ullmann N Mirra V Di Marco A Pavone M Porcaro F Negro V. Asthma: differential diagnosis and comorbidities. Front Pediatr. (2018) 6:276. 10.3389/fped.2018.00276 30338252 PMC6178921

[7] Tay TR Hew M. Comorbid “treatable traits” in difficult asthma: current evidence and clinical evaluation. Allergy. (2018) 73:1369–82. 10.1111/all.13370 29178130

[8] Bardin PG Rangaswamy J Yo SW. Managing comorbid conditions in severe asthma. Med J Aust. (2018) 209:S11–7. 10.5694/mja18.00196 30453867

[9] Ferkh KE Nwaru BI Griffiths C Patel A Sheikh A. Healthcare costs of asthma comorbidities: a systematic review protocol. BMJ Open. (2017) 7:e015102. 10.1136/bmjopen-2016-015102 28566366 PMC5730013

[10] Collaborators, GBDH Global, regional, and national burden of migraine and tension-type headache, 1990–2016: a systematic analysis for the Global Burden of Disease Study. Lancet Neurol. (2018) 17:954–76. 10.1016/S1474-4422(18)30322-3 30353868 PMC6191530

[11] Buse DC Reed ML Fanning KM Bostic R Dodick DW Schwedt TJ. Comorbid and co-occurring conditions in migraine and associated risk of increasing headache pain intensity and headache frequency: results of the migraine in America symptoms and treatment (MAST) study. J Headache Pain. (2020) 21:23. 10.1186/s10194-020-1084-y 32122324 PMC7053108

[12] Hayashi T. Asthma and migraine: is asthma a part of acephalgic migraine? A hypothesis. Ann Allergy. (1988) 60:374. 2451891

[13] Turan MO Susuz ÇÇ Turan PA. Presence of headache and migraine in asthma patients. Turk Thorac J. (2017) 18:47–51. 10.5152/TurkThoracJ.2017.16008 29404159 PMC5783079

[14] Subbarao P Mandhane PJ Sears MR. Asthma: epidemiology, etiology and risk factors. CMAJ. (2009) 181:E181–90. 10.1503/cmaj.080612 19752106 PMC2764772

[15] Martin VT Fanning KM Serrano D Buse DC Reed ML Lipton RB. Asthma is a risk factor for new onset chronic migraine: results from the American migraine prevalence and prevention study. Headache. (2016) 56:118–31. 10.1111/head.12731 26581563

[16] Edginton S O'Sullivan DE King WD Lougheed MD. The effect of acute outdoor air pollution on peak expiratory flow in individuals with asthma: a systematic review and meta-analysis. Environ Res. (2020) 5:110296. 10.1016/j.envres.2020.110296 33031812

[17] Lee H Myung W Cheong HK Yi SM Hong YC Cho SI. Ambient air pollution exposure and risk of migraine: synergistic effect with high temperature. Environ Int. (2018) 121:383–91. 10.1016/j.envint.2018.09.022 30245361

[18] May A Schulte LH. Chronic migraine: risk factors, mechanisms and treatment. Nat Rev Neurol. (2016) 12:455–64. 10.1038/nrneurol.2016.93 27389092

[19] Moher D Liberati A Tetzlaff J Altman DG Group P Preferred reporting items for systematic reviews and meta-analyses: the PRISMA statement. BMJ. (2009) 339:332–6. 10.1136/bmj.b2535 PMC3090117 21603045

[20] Stang A. Critical evaluation of the Newcastle-Ottawa scale for the assessment of the quality of nonrandomized studies in meta-analyses. Eur J Epidemiol. (2010) 25:603–5. 10.1007/s10654-010-9491-z 20652370

[21] Higgins JP Thompson SG Deeks JJ Altman DG. Measuring inconsistency in meta-analyses. BMJ. (2003) 327:557–60. 10.1136/bmj.327.7414.557 12958120 PMC192859

[22] Kim SY Min CY Oh DJ Lim JS Choi HG. Bidirectional association between asthma and migraines in adults: two longitudinal follow-up studies. Sci Rep. (2019) 9:18343. 10.1038/s41598-019-54972-8 31798009 PMC6892888

[23] Davey G Sedgwick P Maier W Visick G Strachan DP Anderson HR. Association between migraine and asthma: matched case–control study. Br J Gen Pract. (2002) 52:723–7. 12236275 PMC1314412

[24] Czerwinski S Gollero J Qiu C Sorensen TK Williams MA. Migraine-asthma comorbidity and risk of hypertensive disorders of pregnancy. J Pregnancy. (2012) 2012:858097. 10.1155/2012/858097 22934185 PMC3425816

[25] Lateef TM Cui L Nelson KB Nakamura EF Merikangas KR. Physical comorbidity of migraine and other headache in United States adolescents. J Pediatr. (2012) 161:308–13. 10.1016/j.jpeds.2012.01.040 22381023 PMC4408276

[26] Wei CC Lin CL Shen TC Chen AC. Children with allergic diseases have an increased subsequent risk of migraine upon reaching school age. J Investig Med. (2018) 66:1064–8. 10.1136/jim-2018-000715 29903897

[27] Peng YH Chen KF Liao WC Hsia TC Chen HJ Yin MC. Association of migraine with asthma risk: a retrospective population-based cohort study. Clin Respir J. (2018) 12:1030–7. 10.1111/crj.12623 28268255

[28] Chen YC Tang CH Ng K Wang SJ Comorbidity profiles of chronic migraine sufferers in a national database in Taiwan. J Headache Pain. (2012) 13:311–9. 10.1007/s10194-012-0447-4 22527034 PMC3356468

[29] Le H Tfelt-Hansen P Russell MB Skytthe A Kyvik KO Olesen J. Co-morbidity of migraine with somatic disease in a large population-based study. Cephalalgia. (2011) 31:43–64. 10.1177/0333102410373159 20974590

[30] Aamodt AH Stovner LJ Langhammer A Hagen K Zwart JA. Is headache related to asthma, hay fever, and chronic bronchitis? The Head-HUNT Study. Headache. (2007) 47:204–12. 10.1111/j.1526-4610.2006.00597.x 17300360

[31] Graif Y Shohat T Machluf Y Farkash R Chaiter Y. Association between asthma and migraine - a cross-sectional study of over 110,000 Adolescents. Clin Respir J. (2018) 12:2491–6. 10.1111/crj.12939 30004178

[32] Becker C Brobert GP Almqvist PM Johansson S Jick SS Meier CR The risk of newly diagnosed asthma in migraineurs with or without previous triptan prescriptions. Headache J Head Face Pain. (2008) 48:606–10. 10.1111/j.1526-4610.2007.01030.x 18194300

[33] Tsiakiris G Neely G Lind N Nordin S. Comorbidity in allergic asthma and allergic rhinitis: functional somatic syndromes. Psychol Health Med. (2017) 22:1163–8. 10.1080/13548506.2016.1276606 28034321

[34] Headache Classification Committee of the International Headache Society Classification and diagnostic criteria for headache disorders, cranial neuralgias and facial pain. Cephalalgia. (2004) 24:1–160. 3048700

[35] Olesen J Headache Classification Committee of the International Headache Society (IHS) the international classification of headache disorders, asbtracts. Cephalalgia. (2018) 38:1–211. 10.1177/0333102417738202 29368949

[36] Sayyah M Saki-Malehi A Javanmardi F Forouzan A Shirbandi K Rahim F Which came first, the risk of migraine or the risk of asthma? A systematic review. Neurol Neurochir Pol. (2018) 52:562–9. 10.1016/j.pjnns.2018.07.004 30119907

[37] Ozge A Uluduz D Bolay H Co-occurrence of migraine and atopy in children and adolescents: myth or a casual relationship? Curr Opin Neurol. (2017) 30:287–91. 10.1097/WCO.0000000000000439 28248699

[38] Aupiais C Wanin S Romanello S Spiri D Moretti R Boizeau P. Association between migraine and atopic diseases in childhood: a potential protective role of anti -allergic drugs. Headache. (2017) 57:612–24. 10.1111/head.13032 28160287

[39] Dirican N Demirci S Cakir M. The relationship between migraine headache and asthma features. Acta Neurol Belgica. (2017) 117:531–6. 10.1007/s13760-017-0764-0 28258562

[40] Tomljenovic D Baudoin T Megla ZB Geber G Scadding G Kalogjera L. Females have stronger neurogenic response than males after non-specific nasal challenge in patients with seasonal allergic rhinitis. Med Hypotheses. (2018) 116:114–8. 10.1016/j.mehy.2018.04.021 29857893

[41] Levy D Burstein R Kainz V Jakubowski M Strassman AM. Mast cell degranulation activates a pain pathway underlying migraine headache. Pain. (2007) 130:166–76. 10.1016/j.pain.2007.03.012 17459586 PMC2045157

[42] Theoharides TC Donelan J Kandere-Grzybowska K Konstantinidou A. The role of mast cells in migraine pathophysiology. Brain Res Brain Res Rev. (2005) 49:65–76. 10.1016/j.brainresrev.2004.11.006 15960987

[43] Galli SJ Tsai M. IgE and mast cells in allergic disease. Nat Med. (2012) 18:693–704. 10.1038/nm.2755 22561833 PMC3597223

[44] Liccardi G Salzillo A Calzetta L Cazzola M Matera MG Rogliani P. Can bronchial asthma with an highly prevalent airway (and systemic) vagal tone be considered an independent asthma phenotype? Possible role of anticholinergics. Respir Med. (2016) 117:150–3. 10.1016/j.rmed.2016.05.026 27492525

[45] Shelukhina I Mikhailov N Abushik P Nurullin L Nikolsky EE Giniatullin R. Cholinergic nociceptive mechanisms in rat meninges and trigeminal ganglia: potential implications for migraine pain. Front Neurol. (2017) 8:163. 10.3389/fneur.2017.00163 28496430 PMC5406407

[46] Kim JH. The emerging role of TRPV1 in airway inflammation. Allergy Asthma Immunol Res. (2018) 10:187–8. 10.4168/aair.2018.10.3.187 29676065 PMC5911437

[47] Meents JE Neeb L Reuter U TRPV1 in migraine pathophysiology. Trends Mol Med. (2010) 16:153–9. 10.1016/j.molmed.2010.02.004 20347391

[48] Benemei S De Cesaris F Fusi C Rossi E Lupi C Geppetti P. TRPA1 and other TRP channels in migraine. J Headache Pain. (2013) 14:1–8. 10.1186/1129-2377-14-71 23941062 PMC3844362

[49] Satia I Nagashima A Usmani OS. Exploring the role of nerves in asthma; insights from the study of cough. Biochem Pharmacol. (2020) 7:113901. 10.1016/j.bcp.2020.113901 32156662

[50] Puebla-Neira D Calhoun WJ. Why do asthma patients cough? New insights into cough in allergic asthma. J Allergy Clin Immunol. (2019) 144 (3):656–7. 10.1016/j.jaci.2019.06.030 31279010

