# Supplementary material for: The Comorbid Relationship Between Migraine and Asthma: A Systematic Review and Meta-Analysis of Population-Based Studies
Source: Front Med (Lausanne). 2021 Jan 13;7:609528. doi: 10.3389/fmed.2020.609528 (PMC7838157; doi:10.3389/fmed.2020.609528)
Supplement: Supplementary file 1 [file Table_1.DOCX]

**Supplementary file 1:** The quality of studies included in systematic review and meta-analysis

| Study name | NOS score | Quality level |
| --- | --- | --- |
| [Buse DC](https://www.ncbi.nlm.nih.gov/pubmed/?term=Buse%20DC%5BAuthor%5D&cauthor=true&cauthor_uid=32122324), 2020 (11) | 6 | Moderate |
| Kim SY, 2019 (22) | 9 | High |
| Wei CC, 2018 (26) | 9 | High |
| Peng YH, 2018 (27) | 9 | High |
| Graif Y, 2018 (31) | 8 | High |
| Tsiakiris G, 2017 (27) | 8 | High |
| Peng YH, 2016 (4) | 9 | High |
| Lateef TM, 2012 (25) | 7 | Moderate |
| Czerwinski S, 2012 (24) | 8 | High |
| Chen YC, 2012 (28) | 7 | Moderate |
| Le H, 2011 (29) | 6 | Moderate |
| Becker C, 2008 (32) | 8 | High |
| Aamodt AH, 2007 (30) | 7 | Moderate |
| Davey G, 2002 (23) | 8 | High |
